# The Evolution of Enzyme Specificity in the Metabolic Replicator Model of Prebiotic Evolution

**DOI:** 10.1371/journal.pone.0020931

**Published:** 2011-06-16

**Authors:** Balázs Könnyű, Tamás Czárán

**Affiliations:** 1 Department of Plant Taxonomy and Ecology, Institute of Biology, Eötvös Loránd University, Budapest, Hungary; 2 Theoretical Biology and Ecology Research Group, Institute of Biology, Eötvös Loránd University, Budapest, Hungary; University of Wyoming, United States of America

## Abstract

The chemical machinery of life must have been catalytic from the outset. Models of the chemical origins have attempted to explain the ecological mechanisms maintaining a minimum necessary diversity of prebiotic replicator enzymes, but little attention has been paid so far to the evolutionary initiation of that diversity. We propose a possible first step in this direction: based on our previous model of a surface-bound metabolic replicator system we try to explain how the adaptive specialization of enzymatic replicator populations might have led to more diverse and more efficient communities of cooperating replicators with two different enzyme activities. The key assumptions of the model are that mutations in the replicator population can lead towards a) both of the two different enzyme specificities in separate replicators: efficient “specialists” or b) a “generalist” replicator type with both enzyme specificities working at less efficiency, or c) a fast-replicating, non-enzymatic “parasite”. We show that under realistic trade-off constraints on the phenotypic effects of these mutations the evolved replicator community will be usually composed of *both types of specialists* and of a limited abundance of *parasites*, provided that the replicators can slowly migrate on the mineral surface. It is only at very weak trade-offs that generalists take over in a phase-transition-like manner. The parasites do not seriously harm the system but can freely mutate, therefore they can be considered as pre-adaptations to later, useful functions that the metabolic system can adopt to increase its own fitness.

## Introduction

The complex, specific and efficient biocatalysts (enzymes) present in all recent forms of life are obviously the products of long Darwinian adaptation, therefore they could not be present on prebiotic Earth. The strongest evolutionary pressure must have affected the spatial structure and the amino acids closest to the active centre of the enzymes, i.e., the details of the peptide structure most responsible for the catalytic function. Maintaining the specific amino acid sequence (primary structure) of an enzyme has evolutionary relevance only as long as it affects its catalytic function. It is mostly the evolutionary “wobbling” of the less important structural elements in distant taxa that explains the divergence of enzymes which are similar in terms of the spatial structure of their active centres and in their biological function (i.e., they catalyse the same reaction), but still have very different amino acid sequences.

Studies of early evolution have long acknowledged the essential role catalysts must have played in the origin of life. It was in Wächtershäuser's hypothesis of the *“prebiotic pizza”* where inorganic compounds were first assumed to carry the function of early biocatalysts [1]–[3]. According to the hypothesis the first chemical reactions ultimately leading to life would have taken place on mineral (e.g., pyrite) surfaces. The prebiotic pizza offers a solution to the thermodynamic problem of condensation reactions yielding water molecules among their products in an environment saturated with water (i.e., in the *“prebiotic soup”*; [4]). The surface of pyrite is also capable of adsorbing organic substrates, thereby increasing their concentrations and catalysing their reactions. Even though this inorganic catalytic effect might have been neither very specific nor very efficient, it might have been sufficient to aid the emergence of the first relatively complex, enzyme-like macromolecule catalysts, which could have worked bound to the surface themselves thereafter [5], [6]. Whether these first catalytic macromolecules were peptides (like most biocatalysts today) or some very different chemical species is still debated [7], [8]. The reason why the first evolvable biocatalysts could not be peptides is that the transmission of the information content of peptides to the next generation would have required a very complicated and very specific cellular apparatus - as a peptide-based genetic machinery - for which there is no evidence in extant organisms [4], [9]. It seems more plausible to assume that the first enzymes were macromolecules produced on mineral surfaces, capable of playing the dual role of the phenotypic function of biocatalysis and that of genetic information transfer, thus evading the difficult problem of the lack of translation at the wake of life. The most likely candidate for this role is the RNA molecule, because it can bind to mineral surfaces due to its electrostatic charges, and its production on mineral surfaces has been proven experimentally [10]–[13]. The nucleobase sequence of RNA stores biologically meaningful information that, in principle, is not difficult to copy, and within-strand Watson-Crick type base-pairing may produce various complicated and reproducible spatial structures which may show very different catalytic activities depending on the base sequence of the molecule (*RNA World*, [14]–[19]). Recent empirical research has provided ample support to this hypothesis by demonstrating the wide spectrum of catalytic activities of RNA, which led to the hypothesis of a surface-bound RNA World [10]–[13], [15], [20], [21].

Of course there remain many – so far unresolved – questions with regard to the chemical mechanisms implied by the RNA World hypothesis. To mention just a few: the synthesis of ribose, phosphates and the four nucleobases under plausible prebiotic environmental conditions is far from straightforward, but in the light of recent empirical work they seem not hopeless either. For example the *formose reaction* (a possible route for ribose synthesis) and the *Stercker-synthesis* (amino acids, nucleobases and many more biologically important compounds) require very different chemical milieu [22]–[25] suggesting that they should have been separated in space if these were to produce ribose and nucleobases for RNA replication. However, recently it was shown that there exists a different chemical route to nucleotides which works at neutral pH and provides an excellent yield [26], [27]. Another notorious problem is that of the concatenation of nucleotides: without activator agents (e.g. imidazole) the proper 3′-5′ direction is not exclusive in the concatenation reaction. Schwartz and his co-workers have shown that phosphites are able to conduct the concatenation of nucleotides in the right direction in water without any activator agent present, and the phosphite would oxidise to phosphate during the reaction [28]. Mineral surfaces also promote the proper 3′-5′ direction of concatenation into long oligonucleotides (40–60-mers) [13], [25] and the homochiral segregation of sugar molecules [29], [30] without which the polymerization process would be blocked by enantiomeric cross-inhibition [31]. von Kiedrowski's suggests that RNA constituents must have been polymerized on mineral surfaces, but many other reactions essential on the way to life could have taken place in the water body of the prebiotic ocean [32].

Yet another problematic issue of the RNA World hypothesis is the “Holy Grail” of prebiotic evolution: the self-replication of RNA molecules, or, more precisely, the lack of an efficient RNA-replicase ribozyme. The problem is connected to *Eigen's paradox* [33] which states in the present context that the precise replication of long ribozymes requires long ribozymes to catalyse their replication. This remains true even if neutral-mutations are considered [34]. There are two possible solutions for the information integration problem: either many short RNA molecules need to cooperate in a compulsory fashion, or an RNA-replicase ribozyme has to evolve somehow “from scratch”, and maintain its own sequence just like that of many other, cooperating ribozymes. Recent experimental results show that both ways are chemically feasible to some extent already. For example, four RNA molecules are shown to cooperate in catalysing their own ligation into a single strand ribozyme [35]–[37]. RNA replicase ribozymes have been seeked for for decades, but it was only very recently that a substantial step forward was taken in that respect: in an *in vitro* evolution project [38] a 189-nucleotide RNA molecule has been discovered that was able to elongate its own 95-nucleotide primer in a template-directed manner. The fidelity of the replication process was 99.4%.

In spite of such empirical “missing links” in connection to the RNA World hypothesis it seems quite probable that the earliest self-reproducing macromolecules with catalytic activities would have been RNA (or very similar) molecules. Theoretical studies suggest that simple (RNA-like) molecules of very weak specificity and efficiency could have evolved to much more specific and efficient enzymatic replicators, through gradual adaptation [39]–[41]. However, due to strict constraints on their spatial structure, the evolution of enzymes is not as simple as assumed in these models. The longer the replicator the more complex its spatial structure will be. This has two important consequences: first, it is not easy to find the “native” conformation of a long macromolecule, and second, a complicated spatial structure makes template-copying more difficult, because long molecules have to be unfolded before being replicated. Such long and complex molecules have a significant fitness handicap at replication compared to shorter and simpler, therefore easy-to-copy ones. However, there is an indirect way to compensate for the direct fitness loss due to structural complexity: complex replicators may have much better catalytic properties. Provided that – due to its catalytic effect – the complex replicator can significantly contribute to the production of its own monomers, its local monomer supply will be higher than that of its simpler, non-catalytic competitors. This indirect fitness advantage may be sufficient to overcompensate the fitness loss caused by decreased replicability, and the catalytic replicator may spread in the population. Of course the above argument applies only to single-step monomer production, i.e., we have to assume that the successful replicator enzyme **A** catalyzes the terminal step of the production of its own monomers – and that all the other resources of replication are present in the environment in sufficient concentrations (i.e., the replicator population is heterotrophic in all other respects). Such an ecological situation is very unlikely to last long: due to increased consumption, some of the substrates of the catalysed reaction will become in short supply as the increasing population of replicator enzyme **A** depletes them, creating selective pressure towards a second enzymatic activity **B** to produce the substrate of replicator **A**, and so on. This Liebig type argument constitutes part of Szathmáry's “progressive sequestration” scenario [42].

How can this second enzymatic activity **B** be obtained by a population of replicators showing enzyme activity **A**? There are essentially two plausible ways to get there: *“enzyme promiscuity”* and *“enzyme cooperation”*. “Enzyme promiscuity” means that a single enzyme is capable of catalyse two (or more) different reactions. This was considered an almost impossible option until very recently, because the dominant view on peptide folding allowed only for a single “native” conformation, and therefore just a single catalytic activity for a peptide enzyme. The conventional view seems to be considerably weakened by now, because quite a few peptide and RNA enzymes have been revealed to admit either more than one native conformations, each of which have to be present to accomplish a single function [43], or more than one native conformations, each with a different function [44]–[47]. Such “multi-functional” enzymes are pre-adapted for later diversification and specialization, to yield different single-functional types, basically by each type gradually losing all but a single activity, and the activity retained becoming more and more specific and efficient. This leads to the second option: the cooperation of two or more different enzymes within the same *“molecular community”*. Coexistence and (co)evolution within such molecular communities have been studied for more than 30 years now, mainly by means of mathematical and simulation models, all of which seek a solution to the basic ecological problem: how could a number of different molecular replicators – using common resources for their replication, therefore being strong competitors of each other – form a stable assembly? All the answers given so far assume some kind of cooperation within the replicator community, to offset the effect of competition which would certainly ruin molecular diversity otherwise. The hypercycle model assumes that the cooperation may be direct, each replicator specifically catalysing the replication of just one other member of the molecular community in a circular topology [40], [33]. Kaufmann's autocatalytic network model [7] also assumes specific and direct catalytic help among different replicators, but not in the rigid circular topology of the hypercycle. According to our metabolic replicator model, the molecular species of the community may be coupled through indirect mutualism, in which case cooperation and coexistence are mediated by a common metabolism which is driven by the replicators as enzymes producing monomers for the community [48], [49]. Whichever approach we take, we need to answer two questions: 1) what makes and keeps the molecular cooperators of the community distinct and specialized, and 2) what saves the community from being invaded and destroyed by parasitic replicators?

We attempt to answer these questions using a simple model of enzymatic speciation in a surface-bound, metabolically coupled replicator population. Each replicator might have two enzyme activities (*E_1_* and *E_2_*, catalysing two different keystone reactions of the metabolism), but this versatility is paid in catalytic efficiency: both enzyme activities of the “generalists” are weak. *E_1_* can be increased by mutation, but only at the expense of *E_2_*, and *vice versa*, i.e., the two enzyme activities are in trade-off. Schultes & Bartel [46] have provided solid empirical proof of the *E_1_*/*E_2_* trade-off for two different folds of the same RNA sequence, each of which had a different catalytic activity: one fold was a ligase, the other catalysed a cleavage reaction. The two enzyme activities have shown strict trade-off: the ligase fold was very weak at cleavage, the cleavage fold was bad at ligation, and the artificial intermediate folds were weak in both activities.

In accordance with the steric constraints on replication mentioned above, we assume that efficient enzyme replicators have a complicated secondary structure which makes them difficult to copy. We assume that replication is a simple, template-directed, non-enzymatic process weakly catalysed by the surface itself. This amounts to postulating that enzyme activities and replication rates (fitnesses) are also traded off: good enzymes make poor templates for replication, and *vice versa*. With these restraints we ask under what circumstances the emergence and the persistence of a community of specialized metabolic replicators can be expected, and to what extent parasitic sequences (fast replicating, short molecules of weak catalytic activity) undermine the efficiency and stability of the metabolic system.

## Methods

The model is a two-dimensional cellular automaton of toroidal lattice topology to avoid edge-effects. Each of the *300×300* square lattice sites may be empty or occupied by a single replicator molecule at any point of time. The basic assumptions with respect to replication and metabolism are similar to those of our earlier Metabolic Replicator Model [48]:

- replicators bind to a mineral surface (represented by the lattice) and they are template-replicated there;
- each replicator has a “basic fitness” parameter, which is its replication rate (*k*) under ideal environmental conditions; this parameter specifies the quality of a replicator - as a template - in its own replication process.
- each replicator is able to catalyze one or two essential reactions of a hypothetical metabolic network;
- each of the two catalytic activities (*E_1_* and *E_2_*) are necessary for metabolism to produce monomers for replication;
- metabolism supplies monomers locally only to sites which have a non-zero sum of enzyme activity in *both E_1_* and *E_2_* within their metabolic neighbourhood (i.e., if any of the two activities is missing from the metabolic neighbourhood, the replicator in the focal site cannot replicate for lack of monomers).
- the random walk (*diffusion*) of replicators is constrained by their adherence to the mineral surface;

To study molecular speciation within the metabolic model, we allow for mutational changes in three traits of the replicators: the two enzyme activities (*E_1_* and *E_2_*) and *k*, the replication rate under ideal conditions (i.e., for a local excess of monomers). The changes in the mutable traits are constrained by a three-way trade-off relation as described later (in 2.3). Based on their mutable traits (*E_1_*, *E_2_* and *k*) replicators can be classified into four different phenotype categories:

1. *specialists:* with a single, strong enzyme activity (either *E_1_* or *E_2_*) and a small replication rate *k*;
2. *generalists:* with two, roughly equal enzyme activities (*E_1_ = E_2_≠*0.0), replication rate depending on the actual values of *E_1_* and *E_2_*, and a small replication rate *k*;
3. *parasites:* replicators without metabolic activity (*E_1_ = E_2_ = *0.0) but of high replication rate;
4. *all the rest:* intermediate phenotypes with different enzymatic activities and replication rates.

With these assumptions we ask which of these phenotype categories evolve in the model, and what determines the actual outcome of the speciation process?

**Figure 1:**
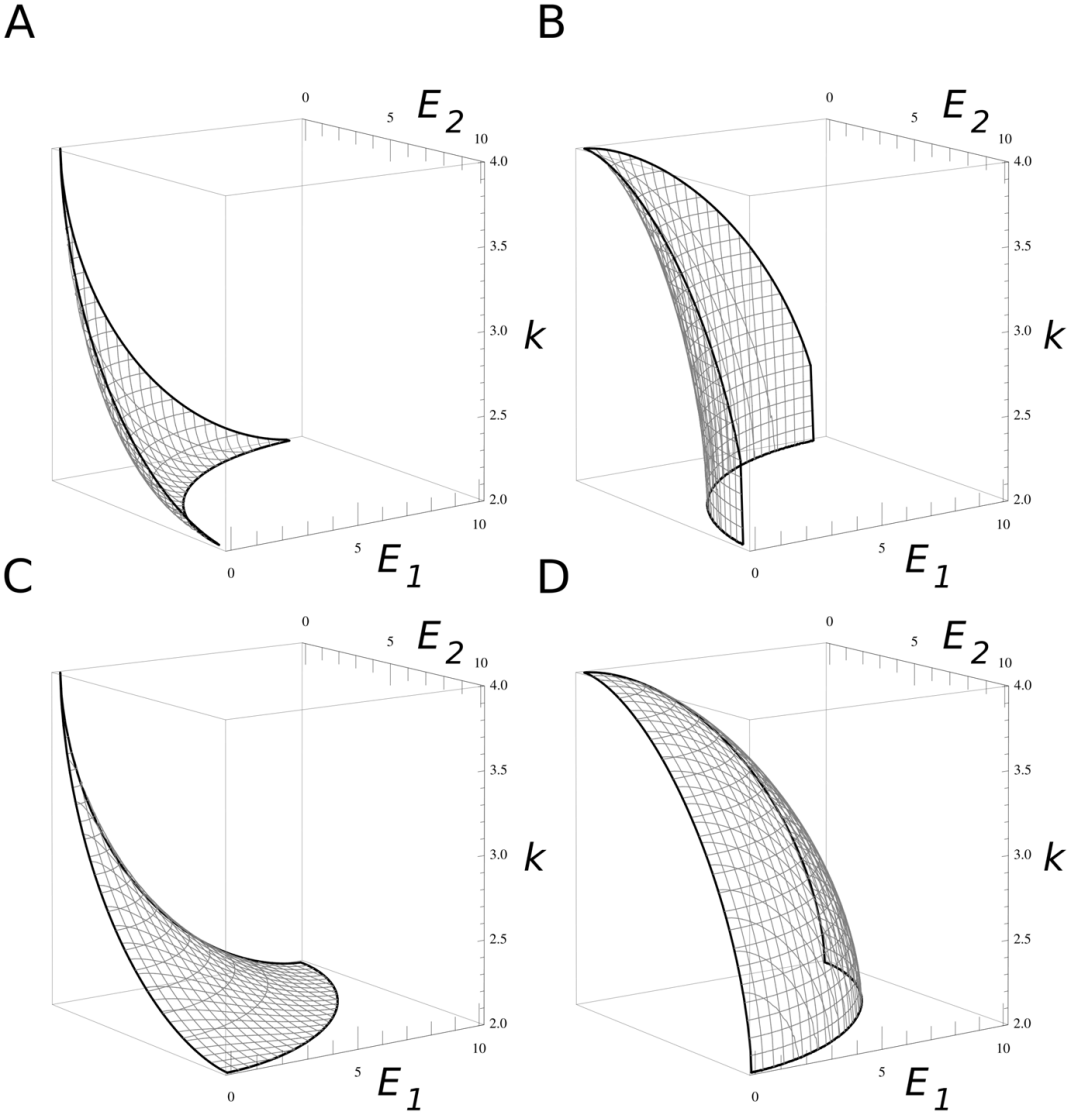
The *E_1_/E_2_/k* trade-off relation. The *E_1_−E_2_−k* trade-off surface as defined by Eq. 6. The trade-off function constrains the phenotypes of newly emerging mutant replicators below the surface. A: convex function representing strong trade-off both between the two enzyme activities *E_1_/E_2_* and between enzyme activities and replication rate, *E/k*. (*b* = 0.6 and *g* = 0.6). B: a function with convex (strong) *E_1_/E_2_* trade-off and concave (weak) *E/k* trade-off (*b* = 0.6 and *g* = 1.67). C: concave (weak) *E_1_/E_2_* and convex (strong) E/k trade-off (*b* = 1.67 and *g* = 0.6). D: both the *E_1_/E_2_* and the *E/k* trade-offs are concave (weak) (*b* = 1.67 and *g* = 1.67). Other parameters: *E_max_* = 10, *k_min_* = 2, *k_max_* = 4.

At *t* = 0, the simulations are initiated with 80% of the sites occupied by replicator molecules, the phenotypes of which are attributed at random (of course with the three-way trade-off constraints considered). We use an asynchronous random updating algorithm to determine the phenotype distribution of the replicator population at the next, (*t*+1)^th^ generation. A schematic representation of the algorithm is given on Figure 1, and the details of the interaction modules are explained below.

### 1. “Death”

Replicators may disappear from the mineral surface for different reasons. They may simply detach and move away from the surface, or they may disintegrate due to chemical “corrosion” (e.g., hydrolysis). Hydrolysis is more frequent in longer molecules, but longer replicators remain adhered to the surface for a longer time. These two effects counteract each other, so we assume that the net dependence of death rates on replicator length is negligible. From the viewpoint of the metabolic system, these events are all “deaths”, and we treat them as such. We assume that deaths occur at a constant rate *p_d_* independent of other traits of the replicator itself and of the other ones in its neighbourhood. If the site being updated contains a molecule, then it disappears with probability *p_d_*, leaving the site empty. We used *p_d_* = 0.1 throughout our simulations.

### 2. Metabolism and competition

If the updated site is empty, then the replicators in its *replication neighbourhood* (i.e., in its von Neumann neighbourhood) compete for the site to put a copy of themselves there. The chance of replicator *i* to win the site is proportional to its actual fitness *W_i_*, which is the product of its replication rate *k_i_* and its local metabolic supply *M_i_*:
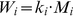
(1)The metabolic supply *M_i_* depends on the two enzyme activities within the *metabolic (Moore) neighbourhood* of replicator *i* in a multiplicative manner:
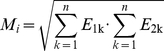
(2)where *n* is the size of the metabolic neighbourhood. *M_i_* is the geometric mean of the local enzyme activities, therefore with any one of the enzyme types missing from the metabolic neighbourhood of *i* the local metabolic supply is zero and replicator *i* cannot be copied.

Even those replicators of which both enzyme activities are non-zero (phenotypes 2. and 4. above) can catalyse only one of the corresponding reactions at a time, since the two different enzyme activities are attributed to two different secondary structures of the molecule. We assume that within the time span of a single generation the replicators do not change conformation, but they can do so between two generations with transition probability
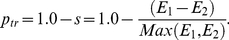
(3)
*s* measures (on the 0.0–1.0 scale) to what extent the replicator in question can be considered as a “specialist”. Eq. 3 assumes that specialist replicators (*s* = 1.0) never change conformation from one generation to the next, whereas “generalists” (of roughly equal enzyme activities for both keystone reactions) almost always do. From the viewpoint of molecular speciation this is not necessarily a realistic assumption; we use it as a worst-case scenario of conformation change from the viewpoint of enzyme specialization: in pure populations of generalist replicators the regular swaps of individual enzyme activities (i.e., of conformation) keeps their metabolism running locally everywhere, whereas purely specialist populations might get stuck in one of the two conformations for a long time, and their local metabolism may break down for lack of the complementary enzyme activity.

The chance of replicator *i* to copy itself onto a neighbouring empty site is
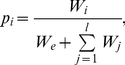
(4)where *j* runs through the replication neighbourhood of the empty site, and *W_e_* quantifies the tendency of a site to remain empty (*W_e_* = 20.0). *W_e_* sets the effect of absolute enzymatic activities in the neighbourhood: weak claims for replication within the replication neighbourhood result in the “no replication” event with a higher probability. Thus the probability that an empty site remains empty is
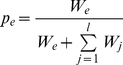
(5)The next state of the empty site is determined by a random draw using the probabilities of Eq's. 4 and 5.

Note that the qualitative behaviour of the model is not affected if we use the *Moore*-type replication neighbourhood (with *l* = 8) instead of the *von Neumann*-type (*l* = 4).

### 3. Mutation and enzyme activity trade-offs

The winner of the local competition for space may be a copy of replicator *i* from within the replication neighbourhood of the empty site. The copy will be “mutated” with probability *p_m_*. The mutant copy will be different from the template with respect to its primary (sequence) and secondary (folding) structures alike, which in turn might have an effect on the phenotype of the offspring, in terms of both enzymatic functionality and replicability. We allow for phenotype changes in three parameters of the replicators: the two enzyme activities (*E_1_* and *E_2_*), and the basic replication rate *k*. As explained in the Introduction, these traits are in a three-way trade-off: efficient enzymes cannot be very good at template replication, and any one of the two enzyme activities can increase only at the expense of the other. These trade-off relations constrain the feasible part of the phenotype space (Figure 1) to under the trade-off constraint surface *C*(*E_1_*, *E_2_*) given by Eq. 6:
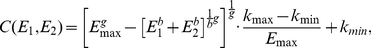
(6)where *E_max_* (*E_max_ = 10.0*) is the absolute maximum of the enzyme activities, *k_max_* (*k_max_* = 2.0 or 2.5 or 4.0 in all simulations) is the highest, *k_min_* (*k_min_* = 2.0) is the lowest possible replication rate of any replicator in the metabolic system, and *b* and *g* determine the strength of the trade-offs which affects the shape of the trade-off surface. This formulation allows us to independently manipulate – through the parameters *b* and *g* – the convexity of the *E_1_−E_2_* and *E−k* trade-off dependencies, respectively. For the enzyme activities, the strict trade-off constraint applies if *E_1_*+*E_2_*<*E_max_*, which in turn means 0.0<*b*<1.0. Then the trade-off function is convex (strong) on the *E_1_*−*E_2_* plane, and the smaller *b* is, the more restrictive the trade-off. *b* = 1.0 represents the limiting case of *E_1_*+*E_2_* = *E_max_*, so that the two enzyme activities together are equivalent to a single specialist's activity. The trade-off is less restrictive for *E_1_*+*E_2_*<2*E_max_*, which translates to 1.0<*b*<∞ and a concave (weak) trade-off function. The upper limit *b* = ∞ represents no trade-off at all: whichever role the enzyme plays, it works as if it were a perfect specialist. The same applies to the trade-off between enzyme activities and replication rates: *g* describes the strength and shape of the trade-off between *E_1_*+*E_2_* and *k*, and the smaller it is, the more convex (stronger) the trade-off relation.

Mutant phenotypes are assigned to the copies of a parent replicator template by a random draw of a point (*E_1_*, *E_2_*, *k*) from below the trade-off surface, so that 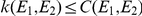, 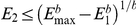, 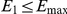. Mutants can be similar or quite different from their parents, which is reasonable to assume for replicator molecules of complex phenotypes: the effects of a mutation on the enzyme activities and the replication rate of an enzymatic replicator are difficult to predict, and they can be of any magnitude, depending on which part of the macromolecular structure is affected. Note that the average mutation is deleterious: selection shifts the phenotype distribution of the replicators towards the trade-off surface (i.e., to higher enzymatic efficiencies and replication rates), but mutants are drawn at random from below the surface.

### 4. Diffusion

Since the replicators are assumed to be bound to the mineral surface by reversible secondary (non-covalent) chemical bonds, their movement on the mineral surface is possible. We have modelled this movement by a simple site-swap algorithm: we choose two neighbouring replicators at random, and swap their positions. The intensity of the resulting diffusion process depends on how many site swaps are allowed per replication (generation) on average, which is defined in the diffusion parameter *D* of the model. Note that even at *D* = 0.0 a minimum of mixing occurs due to the fact that the copy of each template is placed into one of the neighbouring sites during replication, i.e., the offspring moves away from the parent. Again, we have tried two different neighbourhoods for site swapping, and found that choosing neighbours from the von Neumann or the Moore neighbourhood makes no detectable difference in the outcomes of simulation.

## Results

The model predicts the coexistence of multiple replicator species and also enzyme specialization for a large part of its parameter space, even at relatively weak trade-off constraints and limited replicator mobility. Parasites are usually present in the evolved metabolic system at small densities

**Figure 2:**
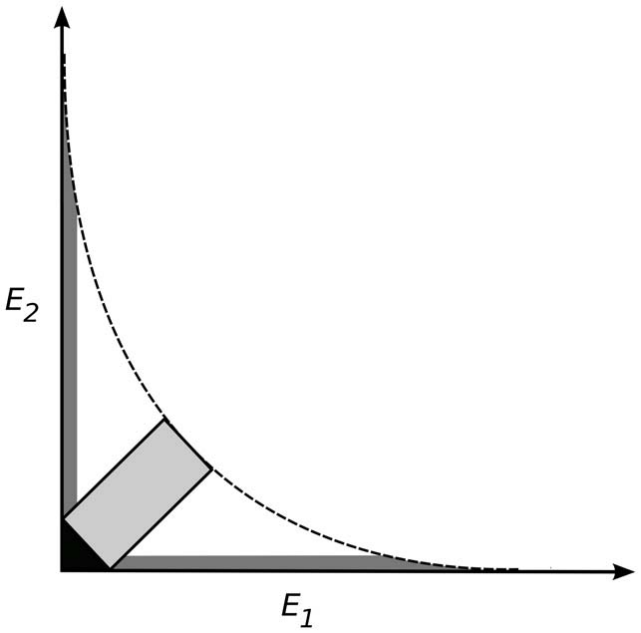
Types of replicators on the *E_1_*−*E_2_* phase plane. *Specialists* (dark grey lines), *generalists* (light grey box), *parasites* (black triangle) and *all the rest* (white). The dashed line represents the *E_1_/E_2_* trade-off relation.

We have screened the effects of changing the crucial model parameters on the outcome of replicator evolution in terms of enzyme specialization and efficiency (i.e., specificity and parasitism). Having run the simulations for sufficiently long times we determined the phenotype distribution of the persistent replicators within the three-dimensional (*E_1_*, *E_2_*, *k_max_*) trait space. Since the trait space under the trade-off constraint surface *C*(*E_1_*,*E_2_*) (Eq. 6) is continuous, it is a matter of definition which of its parts we consider to contain “specialist”, “generalist” and “parasitic” replicator phenotypes. We used the *E_1_*/*E_2_* projection of the three-dimensional trait space to define these phenotype classes. The “absolute” specialists are those replicators which sit on the *E_1_* or the *E_2_* axis of the projected plane, whereas absolute generalists are found on the 45° straight line between these axes. The absolute parasite occupies the origin. Any replicator sufficiently close to these parts of the *E_1_*/*E_2_* plane can be classified as belonging to the corresponding phenotype. Accordingly, we have defined the phenotype categories as shown on Figure 2.

Two of the five relevant model parameters – the shape of the trade-off function of the enzymatic activities (*b*) and the diffusion rate (*D*) – had a strong effect in the sense that changing them across a sufficiently wide range of their possible values results in qualitative changes in the outcomes. The remaining three parameters (the mutation rate *p_m_*, the trade-off shape parameter *g* for replicator enzyme activity and replicability, and the highest possible replication rate *k_max_*) are of much weaker effect. We explain these results below in more detail. Since changing the neighbourhood definition for the replication or the diffusion algorithm did not make substantial differences in the results of simulation, we used the von Neumann neighbourhood for replication and the Moore neighbourhood for diffusion throughout the simulations presented.

### 1. The effects of the trade-off parameters (b and g) and the replication rate (k_max_)

**Figure 3:**
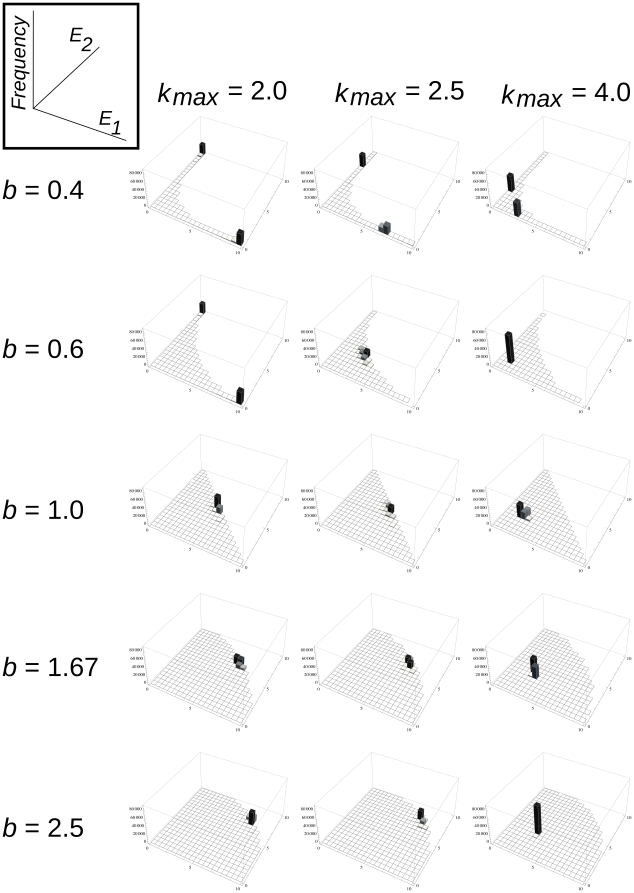
Frequencies of replicator types. A: The frequencies of specialist and generalist replicators as a function of the *b* parameter, at *D* = 0; B: the same, at *D* = 5. Other parameters: *p_m_* = 0.01, *g* = 1.0 and *k_max_* = 2.5 at the 150.000^th^ generation. Note that the frequency of parasitic replicators is less then 1% everywhere in this parameter setting, so we have not plotted it here.

The actual shape of the *E_1_/E_2_* trade-off surface (i.e., the parameter *b*, Figure 1) has an almost all-or-none type effect on the enzyme specialization process: trade-off relations that are convex beyond a certain threshold (for *D* = 0, *k_ma_*_x_ = 2.5 and *g* = 1.0 the threshold is at about *b*∼0.6) yield a high frequency (>80%) of specialists; higher values of *b* result in an overwhelming dominance of generalists, but with their frequency almost completely detached from the actual value of *b* (Figure 3a). This phase-transition-like behavior is preserved at higher replicator mobility as well, but then the threshold of generalist dominance shifts to a very concave shape of the trade-off function: for *D* = 5 it is at *b* = 1.67 (Figure 3b). This means that at somewhat higher replicator mobility specialization pays even if the trade-off between the two enzyme specificities is rather loose (note that as *b* approaches infinity, the trade-off approaches zero).

The evolved enzyme activities and the corresponding (evolved) replication rate of the specialized replicators depend on the *k_max_* parameter: the larger it is, the smaller the actual enzyme activities at equilibrium, because the direct evolutionary advantage of achieving a high replication rate compensates for the loss in the indirect advantage of having a better monomer supply within the metabolic neighbourhood. This leads to the evolutionary shift of the replicators towards a tendency of becoming parasites – the stronger the temptation (i.e., the higher *k_max_*) the closer the replicator community creeps to parasitism. Yet, even at a high advantage of parasitism (*k_max_* = 4.0) the replicators remain enzymatically active and specialized, at least for convex trade-off relations. It is only at very concave *E_1_/E_2_* trade-off functions (i.e., at *b*>1.67 for moderate *D* = 5 replicator mobility) where generalists dominate the replicator community (expect for *g*<1.0 when parasites also appear), but this represents a case of very weak trade-off between the two enzyme activities, therefore it is biochemically unlikely to occur.

We have run a similar series of simulations changing *g*, the *E/k* trade-off parameter as well, with the general conclusion that *g* has a much weaker effect on catalytic specialization than *b*. Its straightforward but moderate and quantitative effect is that very convex *E/k* trade-off functions (very small *g* values) decrease the frequency of generalists and at high mobility it helps the parasites in spreading.

### 2. The effects of diffusion (D)

**Figure 4:**
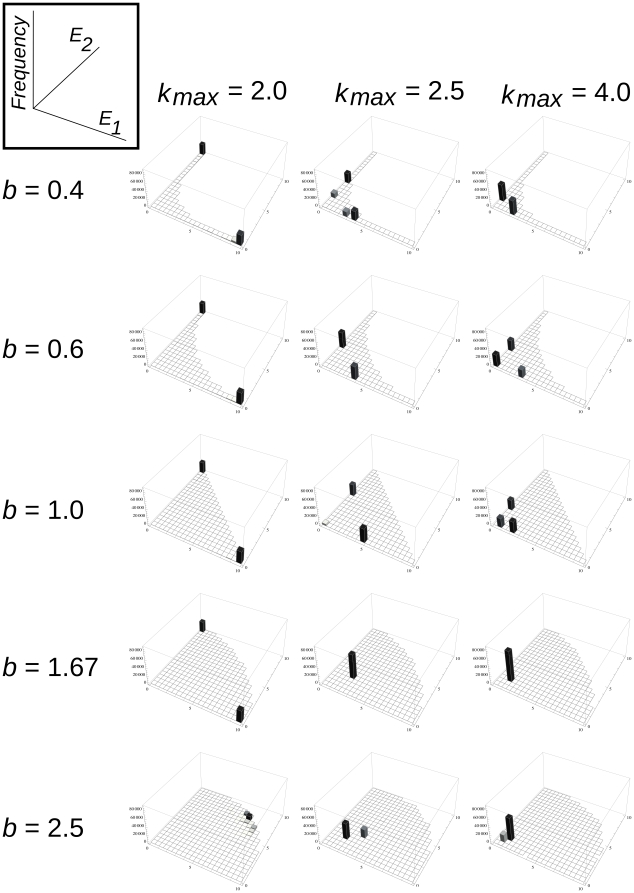
The distribution of enzyme activities without diffusion at different *b* and *k_max_* values. Horizontal axes: enzyme activities *E_1_* and *E_2_*; vertical axis: the frequency of replicators in the lattice, with the corresponding combinations of *E_1_* and *E_2_*.. Other parameters: *p_m_* = 0.01, *D* = 0 and *g* = 1.0, at generation 150.000.

**Figure 5:**
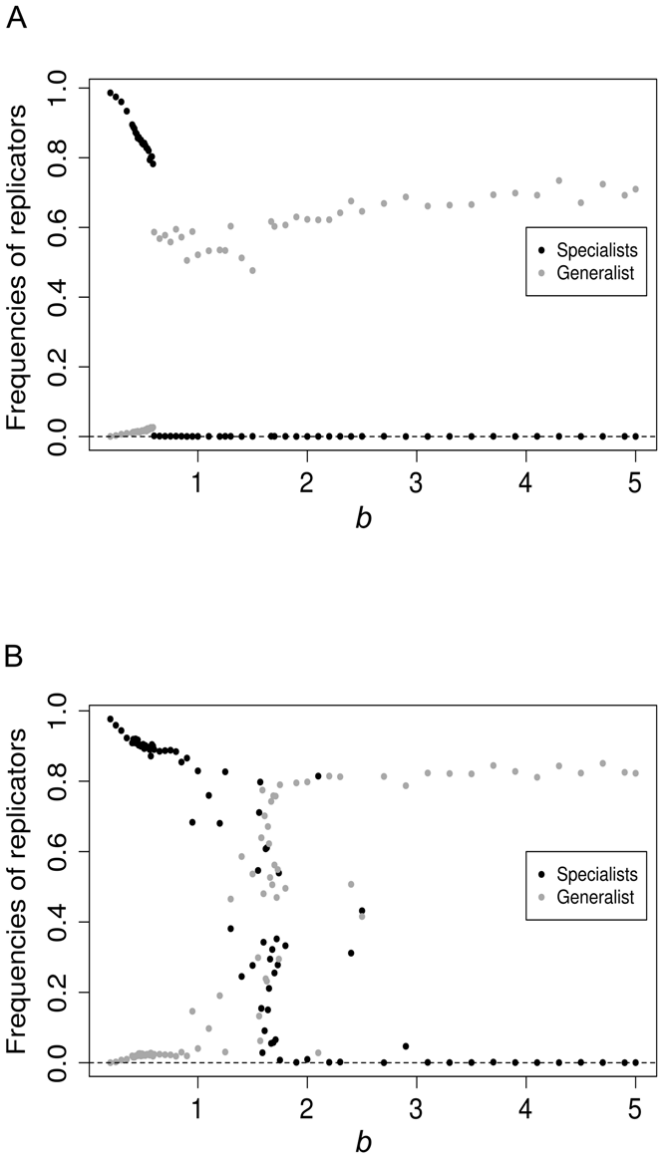
The distribution of enzyme activities with diffusion at different *b* and *k_max_* values. Axes labeled as in Figure 4. Other parameters: *p_m_* = 0.01, *D* = 5 and *g* = 1.0, at generation 150.000.

The mobility of replicators (expressed by the diffusion parameter *D*) has a twofold effect on the efficiency of metabolism, the first aspect of which is positive, and the second one negative (cf. [47]). First, it mixes the different types of enzymatic replicators on the surface, i.e., it dissolves the large, homogeneous patches of replicator clones (consisting of identical ribozymes) which would inevitably arise due to template and copy remaining neighbours in the absence of diffusion. Since the inside of a homogeneous patch of specialized enzymatic replicators lacks the complementary replicators needed to run the metabolism locally, the spatial mixing of specialized replicators is necessary to some extent for the metabolic system to work – metabolism would stop producing monomers and thus replication would be impossible almost everywhere on the surface (except for the borderlines between different patches) otherwise. However, generalist replicators (i.e., those which can switch between different enzyme activities) do not need to be spatially mixed to run local metabolisms, because even clone-mates can complement each other metabolically. This is why generalists almost always exclude specialists at very slow diffusion (*D* = 0.0, Figure 4). (Note that the survival of specialists at *b*≤0.4 is due to the minimal diffusion inherent in the replication algorithm). As the diffusion of replicators becomes faster, specialists become viable and –depending weakly on the shape parameter *b* of the trade-off function between the two enzyme activities – they may win the competition against generalists (Figure 5).

The second effect of increasing replicator mobility is negative for the metabolic system as a whole: parasitic replicators can invade. In a well-mixed population it is the parasitic replicator that has the highest fitness, because it has a high chance of finding itself in a metabolically complete assembly of cooperating replicators which it can exploit then for its own benefit. Since parasites, just as cooperators, can replicate only in a complete metabolic neighbourhood (to which they do not contribute at all), the spatial aggregation of kin replicators, i.e. slow diffusion, prevents the uncontrolled spread of parasites. In fact parasites need to be relatively mobile for taking a sizeable share of abundance within the lattice habitat, because they need to disperse far apart from their clone-mates to be efficient in exploiting the cooperators. Parasite aggregates are doomed to even faster extinction than enzymatic replicator aggregates. As a consequence, at small to moderate rates of diffusion we observe the coexistence of cooperators and parasites.

The balance of cooperators (enzymatic replicators of relatively small rate of population growth *k*) and parasites (replicators of high growth rate with no or very weak enzyme activities *E_1_* and/or *E_2_*) within the replicator community depends on the contribution of two fitness components to population growth. One is the trivial *direct* contribution through the growth parameter *k*, the other is the *indirect* contribution to one's own fitness through the local production of monomers for replication that in turn depends on higher values of *E_1_* and/or *E_2_*. With the possibility of mutational changes in these parameters (which is constrained by the *E*/*k* trade-off relation), the relative weights of the direct and indirect fitness components can be regulated through selection. Obviously, the actual weights of *direct* and *indirect selection* are determined by the diffusion parameter: the higher *D* is, the larger the effect of direct selection relative to that of the indirect one, which means more parasites and less efficient metabolism. This effect is clearly visible on Figs. 3 and 4: the replicator populations approach the origin of the *E_1_/E_2_* trade-off plane and achieve high values of *k* as *D* increases. The “temptation” to become a parasite is the highest if losing some enzyme activity may result in a substantial increase of replication rate, i.e., at high values of *k_max_*.

## Discussion

*In vitro* selection experiments [20], [21] aimed at producing RNA molecules of different phenotypes have suggested that the functional diversity of RNA molecule populations consisting even of rather short digital (nucleotide) sequences might have been sufficiently high for booting up life on prebiotic Earth [5], [6]. It is very likely that, among the many possible functionalities that RNA molecules can possess, some may have evolved to catalyse the copying of the RNA molecule itself and of other RNA molecules, but the template-replicase ribozyme is still to be discovered [17], [50]. Theoretical models [41], [49], [51] have demonstrated that, once some basic functionalities ensuring the self-reproduction of the inhabitants of the RNA world are in place, the way to obtaining more efficient functions (i.e., higher fitness) through Darwinian RNA evolution is open. The resulting communities of early replicators must have evolved towards higher efficiency through cooperation in the long run, but short-term competition among different RNA sequences was obviously inevitable, because the different RNA species must have used the same resources (monomers) for replication. The basically ecological problems of the competitive exclusion of slower replicating RNA sequences and the possible invasion of parasitic ones have been tackled in quite a few theoretical studies [33], [48], [51]–[55]. It is difficult, in many cases impossible, to separate evolutionary and ecological aspects of replicator evolution [56], [57]. This statement applies to the present model as well, in which we have established ecological conditions for the evolution of specialized and efficient enzymatic replicators under different trade-off constraints among two enzyme specificities and the replication rates of metabolically coupled replicators. The trade-off function represents an inseparable relation between the genuine chemical-biochemical constraints on the metabolic roles that replicators can play in the system and the ecological trait of their potential population growth rate *k_max_*.

Increasing enzymatic activity provides better monomer supply for the replicator in its own immediate neighbourhood, therefore it helps its own reproduction by supplying more resources. However, the price of this ecological advantage due to more efficient local catalysis is to be paid by the replicator in terms of its decreased replication rate *k_max_*. This lower replication potential is a direct consequence of the stable and compact secondary structure which is a necessary trait for an efficient catalyst, but obviously makes the replicator more difficult to copy. Parasitic replicators which do not contribute to monomer production but are able to use up the monomer supply faster due to their loose, easily unfoldable and thus easy-to-copy secondary structure are at an ecological advantage compared to the metabolically active enzymatic replicators. Therefore we might expect the parasites to destroy the metabolic system altogether, finally leaving the surface devoid of replicators altogether. Yet, this is not what actually happens. We have found that increasing *k_max_* (i.e., the existence of the *E*/*k_max_* trade-off) results in the evolved replicator population becoming less efficient catalytically but still remaining active and specialized: the majority of the replicators edge closer to the origin of the *E_1_/E_2_* trait plane (i.e., they shift towards parasitism) but they still stay on the specialist axes (cf. Figure 5). This shows that the indirect positive effect of metabolic activity becomes decisive for the dynamics of the system with the frequency of parasites increasing in the replicator population. In all versions of the metabolic model [48], [49] – just as in the present one – it is the indirect metabolic cooperation of the replicators and their limited mobility that makes the system persistent in spite of the differences in replication rates. For the enzymatic replicators to be able to replicate they need their metabolic neighbourhood to be complete. This requires the presence of all *replicator species* within the metabolic neighbourhood in the previous models, and the presence of all *enzyme activities* in this one. In the absence of diffusion there is only one way to maintain all the necessary enzyme activities within a metabolic neighbourhood consisting of identical replicators: all of them must be “generalists” with both enzyme functions present in the same macromolecule – of course at a low activity on both accounts, because this is what the *E_1_/E_2_* trade-off constraint permits. Even very modest replicator diffusion allows for enzymatic replicator specialization, because it mixes different specialized replicator species, so that they can be present together in most metabolic neighbourhoods, allowing for a more efficient local metabolism and, consequently, for the exclusion of less efficient generalists (Figure 5). Our results also suggest that – between rather wide limits – the actual shape of the *E*/*k_max_* trade-off function (the parameter *g*) does not have a decisive effect on enzymatic replicator specialization and the survival of the system. It is only for biochemically infeasible, very concave *E*/*k_max_* trade-off shapes that specialized enzymatic replicators are displaced by generalists.

As we have briefly mentioned in Sect. 3.1., there are *direct* and *indirect* selection effects which influence the evolution of enzymatic functions. Indirect selection favours those functions of the replicators which have a positive influence on their cooperation through which they gain indirect benefits. Under indirect selection the replicators evolve towards becoming more efficient enzymes, the communities of which have more chances to spread due to their better resource supply. Efficient specialists are expected to show up only at a minimum of spatial mixing: replicator mobility helps to harvest indirect selective advantages. Direct selection attempts to maximize the growth rate of the replicators, which pushes the system towards the prevalence of parasites according to the *E*/*k_max_* trade-off constraint. Direct selection is responsible for decreasing the enzyme activities of replicators, and, at the extreme, the dominance of parasites and the collapse of the metabolic system. Real parasites can spread only within metabolically active neighbourhoods, because pure parasite patches produce no resources for replication, and they die out. Therefore, parasites cannot evolve in the absence of diffusion, i.e., at zero replicator mobility. Increased diffusion helps the parasites in finding exploitable neighbourhoods and to avoid the pure-parasite dead end of the dynamics of the system. Under such circumstances parasites coexist with the enzymatic replicators [48], [49]. That is, direct and indirect selection are both enhanced by diffusion [49]; indirect selection helps coexistence, whereas direct selection favours parasitism. We have shown that at a large interval of the *D* parameter axis these two effects are balanced, and the result is a community of more or less specialized and efficient enzymatic replicators, infected by a population of parasites.

Just like in the model of Könnyű and Czárán [49], the controlled presence of the parasite may be even advantageous for the metabolic system as a whole: lacking selective pressures sufficiently effective to completely eliminate it, and also lacking any positive selection to maintain its sequence information, the parasite may freely mutate. Thus it can wander about in the sequence space, and it has a chance to find a function there which is useful for the system as a whole. That is, the parasite is persistent and pre-adapted to many possible beneficial functions, including new metabolic enzyme activities, replicase activity, or membrane production. All these functions must have evolved at some stage of the origin of life, leading to the first membrane-covered macromolecule communities (proto-cells), in which a new and more efficient level of selection described in the stochastic corrector model [53], [57] could have acted to maintain the optimal molecular composition of the earliest living creatures.

## References

[1] Wächtershäuser G (1990). Evolution of the first metabolic cycles.. Proceedings of the National Academy of Sciences of the USA.

[2] Wächtershäuser G (2000). Life as we don't know it.. Science.

[3] Wächtershäuser G (2007). On the chemistry and evolution of the pioneer organism.. Chemistry and Biodiversity.

[4] Maynard-Smith J, Szathmáry E (1995). The major transitions of evolution.

[5] Ma W, Yu C, Zhang W, Hu J (2007). Nucleotide synthetase ribozymes may have emerged first in the RNA world.. RNA.

[6] Garay J (2010). Active centrum hypothesis: the origin of chiral homogenity and the RNA-World.. Bio Systems.

[7] Kauffman S (1986). Autocatlytic sets of proteins.. Journal of Theoretical Biology.

[8] Nelson KE, Levy M, Miller SL (2000). Peptide nucleic acids rather than RNA may have been the firs genetic molecule.. Proceedings of the National Academy of Sciences of the USA.

[9] Orgel LE (2003). Some consequences of the RNA world hypothesis.. Origins of Life and Evolution of Biospheres.

[10] Ferris JP, Hill RA, Liu R, Orgel LE (1996). Synthesis of long prebiotic oligomers on mineral surfaces.. Nature.

[11] Franchi M, Ferris JP, Gallori E (2003). Cations as mediators of the adsorption of nuclec acids on clay surfaces in prebiotic environments.. Origins of Life and Evolution of Biospheres.

[12] Franchi M, Gallori E (2005). A surface-mediated origin of RNA World: biogenic activities of clay-absorbed RNA molecules.. Gene.

[13] Ferris JP (2006). Montmorillonite-catalysed formation of RNA oligomers: the possible role of catalysis in the origins of life.. Philosohical Transactions of the Royal Society B.

[14] Gilbert W (1986). Origin of life: the RNA world.. Nature.

[15] Joyce GF (2002). The antiquity of RNA-based evolution.. Nature.

[16] Lilley DMJ (2003). The origins of RNA catalysis in ribozyme.. Trends in Biochemical Sciences.

[17] Chen X, Li N, Ellington AD (2007). Ribozyme catalysis of metabolism in the RNA World.. Chemistry & Biodiversity.

[18] Cech TR (2009). Crawling out of the RNA World.. Cell.

[19] McConnell TS, Hercchlag D, Cech TR (1997). Effects of divalent metal ions on individual steps of the Tetrahymena ribozyme reaction.. Biochemistry.

[20] Landweber LF, Simon PJ, Wagner TA (1998). Ribozyme engineering and early evolution.. Bio Science.

[21] Bartel DP, Unrau PS (1999). Constructing an RNA world.. Trends in Genetics.

[22] Ferris JP, Joshi PC, Edelson EH, Lawless JG (1978). HCN: A plausible source of purines, pyrimidines and amino acids on the primitive Earth.. Journal of Molecular Evolution.

[23] Ferris JP, Hagan JR (1984). HCN and chemical evolution: the possible role of cyano compounds in prebiotic synthesis.. Tetrahedron.

[24] Weber AL (2008). Sugar-driven prebiotic sythesis of 3,5(6)-dimethylpyrazin-2-one: a possible nucleobase of a primitve replication process.. Origins of Life and Evolution of Biospheres.

[25] Robertson MP, Joyce GF (2010). The origins of the RNA world.. Cold Spring Harbor Perspectives in Biology.

[26] Powner MW, Gerland B, Sutherland JD (2009). Synthesis of activated pyrimidine ribonucleotides in prebiotic plausible conditions.. Nature.

[27] Powner MW, Sutherland JD, Szostak JW (2010). Chemoselective multicomponent one-pot assembly of purine precursors in water.. Journal of American Chemical Society.

[28] Schwartz AW (2006). Phosphorus in prebiotic chemistry.. Philosohical Transactions of the Royal Society B.

[29] Bielski R, Tencer M (2007). A possible path to the RNA World: enantioselective and diastereoselective purification of ribose.. Origins of Life and Evolution of Biospheres.

[30] Hazen RM, Filley TR, Goodfriend GA (2001). Selective adsorption of L- and D-amino acids on calcite: implications for biochemical homochirality.. Proceedings of the National Academy of Sciences of the USA.

[31] Joyce GF, Visser GM, van Boeckel CA, van Boom JH, Orgel LE (1984). Chiral selection in poly(C)-directed synthesis of oligo(G).. Nature.

[32] von Kiedrowski G (1996). Priomrdial soup or creps.. Nature.

[33] Eigen M, Schuster P (1979). The Hypercycle.

[34] Kun Á, Santos M, Szathmáry E (2005). Real ribozymes suggest a relaxed error threshold.. Nature Genetics.

[35] Riley CA, Lehman N (2003). Generalized RNA-directed recombination of RNA.. Chamistry & Biology.

[36] Hayden EJ, Lehman N (2006). Self-assemblyof a group I intron from inactive oligonucleotide fragments.. Chamistry & Biology.

[37] Burton AS, Lehman N (2010). Enhancing the prebiotic relevance of a set of covalently self-assembling autorecombining RNAs through in vitro selection.. Journal of Molecular Evolution.

[38] Wochner A, Attwater J, Coulson A, Holliger P (2011). Ribozyme-catalyzed transcription of an active ribozyme.. Science.

[39] Kacser H, Beeby R (1984). Evolution of catalytic proteins or On the origin of enzyme species by means of natural selection.. Journal of Molecular Evolution.

[40] Scheuring I (2000). Avoiding catch-22 of early evolution by stepwise increase in copy fidelity.. Selection.

[41] Szabó P, Scheuring I, Czárán T, Szathmáry E (2002). In silico simulations reveal that replicators with limited dispersal evolve towards higher efficiency and fidelity.. Nature.

[42] Szathmáry E (2007). Coevolution of metabolic networks and membranes: the scenario of progressive sequestration.. Philosohical Transactions of the Royal Society B.

[43] Huang Z, Pei W, Han Y, Jayaseelan S, Shekhtman A (2009). One RNA aptamer sequence, two structures a collaborating pair that inhibits AMPA receptors.. Nucleid Acids Research.

[44] O'Brien PJ, Herschlag D (1999). Catalytic promiscuity and the evolution of new enzymatic activities.. Chemistry and Biology.

[45] Ancel WL, Fontana W (2000). Plasticity, evolvability, and modularity in RNA.. Journal of Experimental Zoology (Mol. Dev. Evol.).

[46] Schultes EA, Bartel DP (2000). One sequence, two ribozymes: implication for the emergence of new ribozyme fold.. Science.

[47] Khersonsky O, Roodveldt C, Tawfik DS (2006). Enzyme promiscuity, evolutionary and mechanistic aspects.. Current Opinion in Chemical Biology.

[48] Czárán T, Szathmáry E, U D, R L, J MJA (2000). Coexistence of replicators in prebiotic evolution.. The Geometry of Ecological Interactions, IIASA and Cambridge University Press.

[49] Könnyű B, Czárán T, Szathmáry E (2008). Prebiotic replicase evolution in a surface-bound metabolic system: parasites as a source of adaptive evolution.. BMC Evol Biol.

[50] McGinness K, Joyce GF (2003). In search of an RNA replicase rybozyme.. Chemistry and Biology.

[51] Scheuring I, Czárán T, Szabó P, Károlyi Gy, Toroczkai Z (2003). Spatial models of prebiotic evolution: soup before pizza?. Origins of Life and Evolution of Biospheres.

[52] Maynard-Smith J (1979). Hypercycles and the origin of life.. Nature.

[53] Szathmáry E, Demeter L (1987). Group selection of early replicators and the origin of life.. Journal of Theoretical Biology.

[54] Boerljis C, Hogeweg P (1991). Spiral wave structure in pre-biotic evolution: hypercicles stable against parasite.. Physica D.

[55] Károlyi Gy, Péntek I, Scheuring I, Tél T, Toroczkai Z (2000). The antiquity of RNA-based evolution.. Proceedings of the National Academy of Sciences of the USA.

[56] Meszena G, Szathmáry E (2001). Adaptive dinamics of parabolic replicators.. Selection.

[57] Zintzaras E, Santos M, Szathmáry E (2002). Living under the challenge of information decay: the stochastic corrector model vs. hypercycles.. Journal of Theoretical Biology.

